# Natural Language Processing framework for identifying abdominal aortic aneurysm repairs using unstructured electronic health records

**DOI:** 10.1038/s41598-025-11870-6

**Published:** 2025-07-21

**Authors:** Daniel C. Thompson, Reza Mofidi

**Affiliations:** 1 Vascular Surgery Specialty Training, Health Education England North East, Newcastle upon Tyne, UK; 2 Academic Department of Military Surgery & Trauma, Royal Centre for Defence Medicine, Birmingham, UK; 3 Department of Vascular Surgery, James Cook University Hospital, Middlesbrough, UK

**Keywords:** Natural Language Processing, NLP, Abdominal aortic aneurysm repair, AAA repair, Electronic health records, National vascular registry, Health care, Health policy, Health services

## Abstract

Patient identification for national registries often relies upon clinician recognition of cases or retrospective searches using potentially inaccurate clinical codes, leading to incomplete data capture and inefficiencies. Natural Language Processing (NLP) offers a promising solution by automating analysis of electronic health records (EHRs). This study aimed to develop NLP models for identifying and classifying abdominal aortic aneurysm (AAA) repairs from unstructured EHRs, demonstrating a proof-of-concept for automated patient identification in registries like the National Vascular Registry. Using the MIMIC-IV-Note dataset, a multi-tiered approach was developed to identify vascular patients (Task 1), AAA repairs (Task 2), and classify repairs as primary or revision (Task 3). Four NLP models were trained and evaluated using 4870 annotated records: scispaCy, BERT-base, Bio-clinicalBERT, and a scispaCy/Bio-clinicalBERT ensemble. Models were compared using accuracy, precision, recall, F1-score, and area under the receiver operating characteristic curve (AUC). The scispaCy model demonstrated the fastest training (2 min/epoch) and inference times (2.87 samples/sec). For Task 1, scispaCy and ensemble models achieved the highest accuracy (0.97). In Task 2, all models performed exceptionally well, with ensemble, scispaCy, and Bio-clinicalBERT models achieving 0.99 accuracy and 1.00 AUC. For Task 3, Bio-clinicalBERT and the ensemble model achieved an AUC of 1.00, with Bio-clinicalBERT displaying the best overall accuracy (0.98). This study demonstrates that NLP models can accurately identify and classify AAA repair cases from unstructured EHRs, suggesting significant potential for automating patient identification in vascular surgery and other medical registries, reducing administra

**Supplementary Information:**

The online version contains supplementary material available at 10.1038/s41598-025-11870-6.

## Introduction

The integration of Natural Language Processing (NLP) in vascular surgery has gained increasing interest, due to its ability to help analyse data from electronic health records (EHRs), provide feedback on current practice and develop support systems to optimise care^1^. NLP techniques have evolved significantly over the years. Initially, text classification tasks relied on rule-based approaches, which, despite being somewhat effective, faced limitations in managing the complexity and variability of natural language, particularly in the biomedical field^2^. These techniques have since evolved from simple heuristic-based models to sophisticated neural network-based architectures^3^. The integration of word embeddings, contextualised embeddings, and transfer learning has significantly enhanced the capability of models to understand and classify text accurately^3^.

The application of domain-specific NLP models, such as scispaCy, has further improved the accuracy and relevance of text classification in specialised fields like biomedical research and clinical practice^4^. ScispaCy, a specialised extension of the spaCy NLP library, is tailored for scientific and clinical text, making it highly effective for tasks that require an understanding of domain-specific terminology and contexts^4,5^.

A major breakthrough in NLP came with the development of transformer-based models, leading to the rapid rise of large language models (LLMs)^6^. Among these, Generative Pre-trained Transformer (GPT) models, such as ChatGPT, have gained widespread recognition for their autoregressive capabilities^7^. In contrast, Bidirectional Encoder Representations from Transformers (BERT) models, developed by Google, can understand the context of words in both directions, offering a different and often superior approach for NLP tasks^8^. These foundational models require extensive pre-training on large text corpora and subsequent fine-tuning on specific tasks using labelled datasets to achieve high performance in domains like text classification, named entity recognition, and span categorisation. Domain-specific adaptations, such as Bio-clinicalBERT for medical texts, further enhance their utility in specialised fields by using transfer learning to improve their applicability and accuracy in various biomedical NLP tasks^9–12^.

Currently, the UK-based National Vascular Registry (NVR) gathers national data on primary AAA repairs, revision AAA repairs, lower limb bypass, lower limb angioplasty, major limb amputation, and carotid endarterectomy. The registry relies on the manual entry of patient data, which is not only time-consuming but also necessitates manual tracking for follow-up data such as outcomes at 30 days post-procedure. The adoption of NLP technologies could significantly reduce this burden by automating data extraction and analysis processes^1,13^.

In vascular surgery, NLP applications have demonstrated significant potential. One such use has been the identification of AAA diagnoses from radiology reports streamlining the process for surveillance or referral initiation^14,15^. AAA features such as maximal diameter within the reports have also been extracted to guide the next management steps^16^. NLP has also shown the potential to be used as a decision support system for predicting aortic dissection, alerting emergency department doctors for appropriate investigation and management with a reported AUC of 0.90^17^. Beyond aortas, studies have demonstrated the use of NLP to more accurately identify patients with peripheral artery disease (PAD) from EHRs and radiology reports^18,19^. Further models have been developed to identify complications such as chronic limb threatening ischaemia^20^. For carotid stenosis, NLP has been used to categorise stenosis severity from imaging reports, achieving a positive predictive value (PPV) of 99% for ultrasound and 96.5% for CTA and MRA^21^. However, there is no published literature of NLP models trained to identify patients who have undergone AAA repairs nor whether they have undergone a primary or revision repair. The differentiation between primary and revision AAA repairs is of clinical importance due to there being two separate forms for NVR data collection of those undergoing primary or revision AAA repairs.

This paper outlines a framework for the development and comparison of four NLP models: a fine-tuned BERT-base LLM, a fine-tuned biomedical domain-specific BERT LLM (Bio-clinicalBERT), a biomedical SpaCY based model (scispaCy) and a Bio-clinicalBERT/scispaCy ensemble model to identify and classify abdominal aortic aneurysm (AAA) repair cases from unstructured free-text EHRs into categories of primary repair and revision repair.

## Methods

### Data collection and preparation

The publicly available MIMIC-IV-Note dataset was used, comprising 331,794 de-identified discharge summaries from 146,815 patients admitted to the Beth Israel Deaconess Medical Center in Boston, MA, USA between 2008 and 2022^22^. The data were unstructured, free text clinical notes containing discharge summaries detailing admission clerking, observations, hospital course, pertinent radiology reports, blood test results, and relevant discharge instructions. All records underwent de-identification in compliance with the Health Insurance Portability and Accountability Act (HIPAA) Safe Harbor guidelines.

To simulate a real-life clinical utility whilst maintaining data privacy, the dataset was pseudo-anonymised using the Python Faker library. Identifiable information placeholders, such as names, unit numbers, dates of birth, admission and discharge dates, and attending physician names, were replaced with realistic fake data. Anonymised names and dates in the clinical narrative were not synthetically generated and a de-identification placeholder remained in place.

### Annotation

The data annotation process was conducted using Prodigy annotation software (Explosive AI, Berlin, Germany), by a Vascular Surgery Specialty Registrar. For Task 1, records for annotation were selected from the full dataset using terms related to vascular surgery as ‘seeds’ for annotation, and an active learning approach was implemented utilising SciSpacy’s en_core_sci_md model. For Tasks 2 and 3, records for annotation were selected from a pool of records extracted using trained models from previous tasks, with additional, more specific seed terms relating to AAA repair to improve annotation efficiency and mitigate class imbalance, as described in the Multi-Tiered Classification Approach below. Each task was annotated independently without inheriting labels from previous tasks. Seed terms can be found in Appendix 1.

For Task 1, EHRs were categorised as ‘Vascular’ if there was a pathology relevant to vascular surgery during the patients’ admission as per National Health Service (NHS) England Service Specifications for Vascular Services^23^. Diabetic foot infections requiring debridement ± revascularisation were also included. Stable chronic vascular conditions not being actively treated during the admission were annotated as ‘Non-vascular’. For Task 2, EHRs were categorised as ‘AAA repair’ if there was a repair of a thoracic and/or abdominal aortic and/or iliac aneurysm during the admission. Isolated ascending aortic aneurysm repairs were excluded. For Task 3, EHRs were categorised as ‘Primary AAA repair’ if a repair occurred on a previously untreated segment of the thoracic and/or abdominal aorta. ‘Revision AAA repair’ was defined as a further repair on a previously treated thoracic and/or abdominal aortic segment such as endoleak or aneurysmal disease at, or adjacent to, the anastomosis/stent landing zone site.

A training curve function was utilised for each annotated dataset to determine the quality of the collected annotations, and whether more training examples would improve accuracy. Annotation was deemed sufficient if a training curve stopped showing improvement in the last 25% of the dataset. Annotated data were split into 80% for training and 20% for evaluation.

### Multi-tiered classification approach

**Fig. 1:**
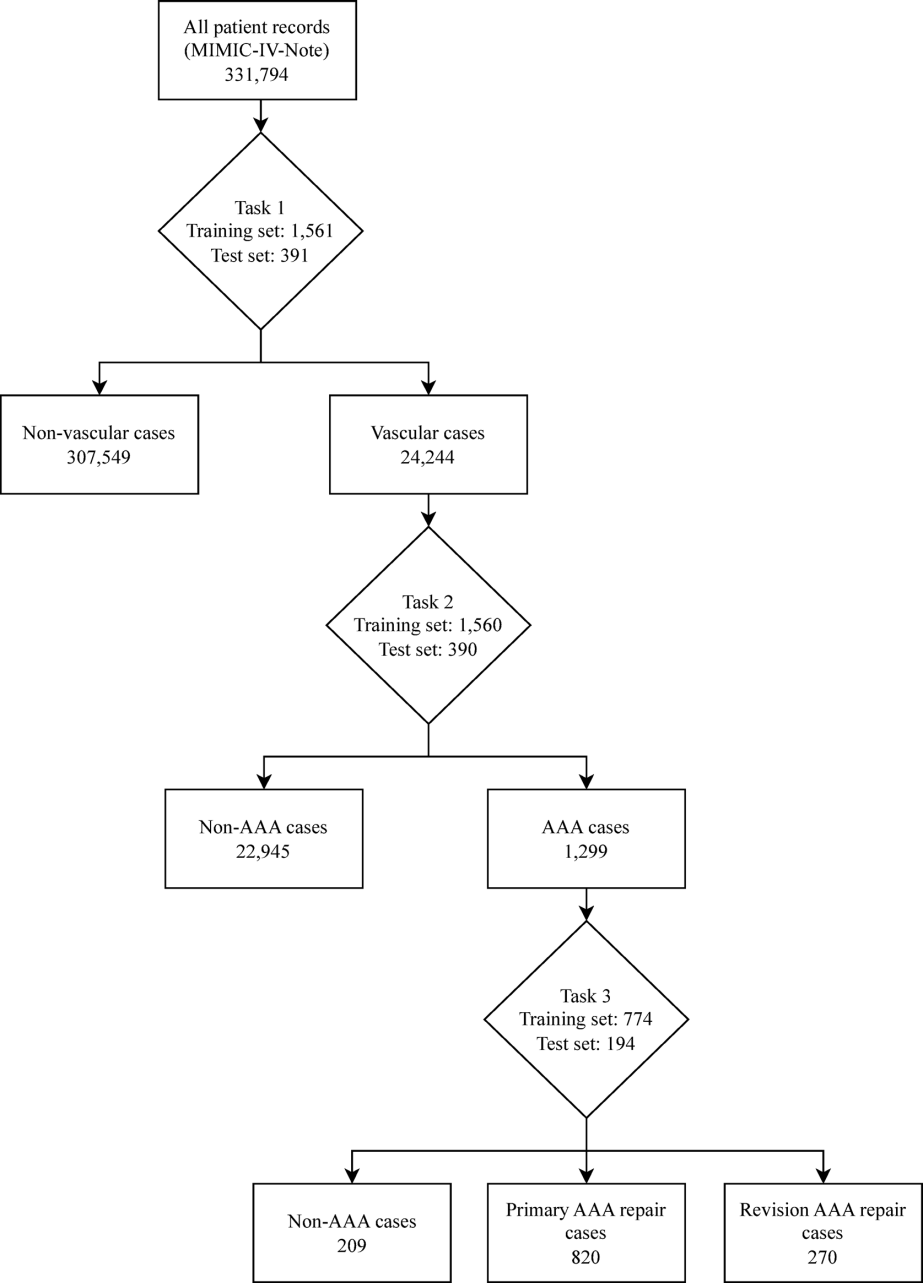
Flowchart showing the extraction of cases using a trained scispaCy model for each classification task. Task 1 - Vascular vs. Non-Vascular classification performed using a threshold of 0.2. Task 2 - AAA vs. Non-AAA classification performed using a threshold of 0.5. Task 3 – Primary AAA repair vs. Revision AAA repair vs. Non-AAA classification performed using a threshold of 0.5. AAA – Abdominal aortic aneurysm.

The classification model used a structured multi-tiered approach using three classification tasks to achieve the aim of classifying AAA repairs, as outlined in the annotation process above. For Task 1, candidate records were initially selected using seed terms related to vascular surgery, then a model was trained to identify vascular surgery related admissions. Each model generates probability scores for class predictions, requiring probability thresholds to determine final classifications. The model was then used to extract vascular-related admissions from the dataset using a probability threshold of 0.2 selected by threshold analysis to maximise recall and capture the broadest possible set of potentially vascular cases for subsequent annotation. This subset dataset was independently annotated for AAA repair cases (Task 2) which was then used to train a model and extract AAA repair records from the vascular dataset using a threshold of 0.5 selected after threshold optimisation analysis. A further model (Task 3) was trained to classify these AAA cases into primary and revision repairs after independent annotation. Task 3 also included a ‘Non-AAA’ classification to be able to appropriately categorise cases that had been misidentified in previous steps. In this paper, the scispaCy model was used to extract the data for each task as demonstrated in Figure 1.

1. **Task 1 and Task 2**: Models were fine-tuned using annotated datasets with two labels:
  - Task 1- Vascular or Non-Vascular.
  - Task 2 - AAA repair or Non-AAA repair.
2. **Task 3**: Model was fine-tuned using an annotated dataset with three labels. Non-AAA cases were included in this classification as previous models were not 100% accurate and some Non-AAA cases remained within the dataset.
  - Task 3 - Primary AAA Repair or Revision AAA Repair or Non-AAA.

### Model architecture

Four NLP models were trained and evaluated: BERT-base^8^, a general-purpose transformer model; Bio-ClinicalBERT^12^, a biomedical domain-specific BERT model pre-trained on clinical texts; scispaCy^4^, a biomedical-focused model from the open-source spaCy NLP framework; and an ensemble model combining scispaCy and Bio-clinicalBERT.

### SpaCy model training

The ‘en_core_sci_md’ model from scispaCy was used to develop an NLP pipeline^4^. The pipeline used ‘tok2vec’ tokenization and ‘textcat_multilabel’ components with other components frozen for training. The training process was configured with a dropout rate of 0.1, Adam optimizer with a learning rate of 0.001, and L2 weight decay. Training was performed for 20 epochs with validation performance monitoring and automatic best model checkpoint selection to ensure optimal model retention. Training was performed using an Apple MacBook M2 Pro (California, USA) 16-core Graphics Processing Unit (GPU).

### BERT model training

#### Tokenization and sliding window approach

Given the varying lengths of medical records, a sliding window tokenization approach was used to overcome the 512 maximum token sequence length constraint of BERT models. To accommodate BERT’s special tokens [CLS] and [SEP], the actual token window was set to 510 tokens. The BertTokenizer from the Hugging Face Transformers library was used for tokenizing the input texts. A stride of 255 tokens was used to allow sufficient overlap between segments, preserving contextual information across the segmented texts.

The segments were converted into model inputs, and probabilities for each segment were predicted using the BERT based models. Softmax function was applied to convert logits into probabilities, ensuring each class probability was normalised. Segment probabilities were then averaged across all segments of the record to obtain aggregated probabilities. A default threshold of 0.5 was applied to these aggregated probabilities to implement classification.

#### Model training

The fine-tuning process was conducted using a Google Colaboratory (California, USA) Tensor Processing Unit (TPU) runtime environment using TensorFlow’s TPU strategy.

The models were compiled with the Adam optimizer. Sparse Categorical Crossentropy loss and accuracy were used as training evaluation metrics. Early stopping (patience = 2) and learning rate reduction on plateau callbacks were implemented to prevent overfitting and dynamically adjust the learning rate, with automatic restoration of best model weights based on validation performance.

The training process used a maximum of 5 epochs, with a batch size of 16. Training terminated when validation loss did not improve for 2 consecutive epochs, with the best-performing model checkpoint automatically restored to ensure optimal model selection.

### Ensemble model

An ensemble model was constructed by combining scispaCy and Bio-clinicalBERT predictions to combine the strengths of different model architectures trained on biomedical text. The ensemble approach aimed to improve classification robustness by combining predictions from models with complementary approaches to clinical language understanding.

Predictions from both trained scispaCy and fine-tuned Bio-clinicalBERT models were integrated into a single pipeline. Predictions from the fine-tuned Bio-clinicalBERT model were obtained by averaging the softmax probabilities across all segmented texts. Predictions from the scispaCy model, configured with a multi-label text categorisation component (‘textcat_multilabel’), were collected and processed. The aggregated probabilities from both models were then combined by averaging to form a combined prediction output.

### Evaluation metrics

For performance evaluation, SciSpaCy, BERT-base and Bio-clinicalBERT and ensemble models were evaluated using accuracy, precision, recall and F1-score calculations per class using a probability threshold of 0.5 to ensure consistent comparison across all models. ROC curves were calculated with AUC values to assess class discrimination based on predicted probabilities.

## Results

A total of 4870 admission records were annotated with 1952 records annotated as vascular or non-vascular for Task 1, 1950 records annotated as AAA repair or non AAA repair for Task 2, and 968 records annotated as primary AAA, revision AAA repair or non-AAA repair for Task 3.

Model training and inference times for BERT and scispaCy models are summarised in Table 1. The calculated average training time across the 3 tasks was 3.03 min/epoch for BERT-base, 2.75 min/epoch for Bio-clinicalBERT and 2 min/epoch for scispaCy. Inference time for scispaCy model was faster than BERT models with a calculated average throughput of 2.87 samples/secs compared with 2.28 samples/secs for BERT-base and 2.32 samples/secs for Bio-clinicalBERT.

**Table 1:** Comparison of training and inference metrics for BERT and scispacy models for task 1 (Vascular vs. Non-Vascular classification), task 2 (AAA vs. Non-AAA classification) and task 3 (Primary AAA repair vs. Revision AAA repair vs. Non-AAA classification). Bio-clinicalBERT and BERT-base fine-tuned using Google Colab tensor processing unit v2. ScispaCy trained using Apple MacBook M2 pro 16-core graphics processing unit (GPU). All inference performed using an Nvidia Tesla L4 GPU.

	Task 1 (*n* = 1952)				Task 2 (*n* = 1950)				Task 3 (*n* = 968)			
	BERT-base	Bio-clinical BERT	scispaCy	Ensemble	BERT-base	Bio-clinical BERT	scispaCy	Ensemble	BERT-base	Bio-clinical BERT	scispaCy	Ensemble
Annotated data for training	1561	1561	1561	1561	1560	1560	1560	1560	774	774	774	774
Annotated data for evaluation	391	391	391	391	390	390	390	390	194	194	194	194
Number of epochs for training	4	3	20	–	4	4	20	–	3	3	20	–
Fine-Tuning Time (mins per epoch)	3.5	2.66	2	–	3.25	3.25	2	–	2.33	2.33	2	–
Inference Time (Total, secs)	186	199	153	347	181	163	133	296	75	74	62	136
Inference Time (secs/sample)	0.476	0.509	0.391	0.887	0.464	0.418	0.341	0.759	0.387	0.381	0.320	0.701
Throughput (samples/secs)	2.10	1.96	2.56	1.13	2.15	2.39	2.93	1.32	2.59	2.62	3.13	1.43

**Fig. 2:**
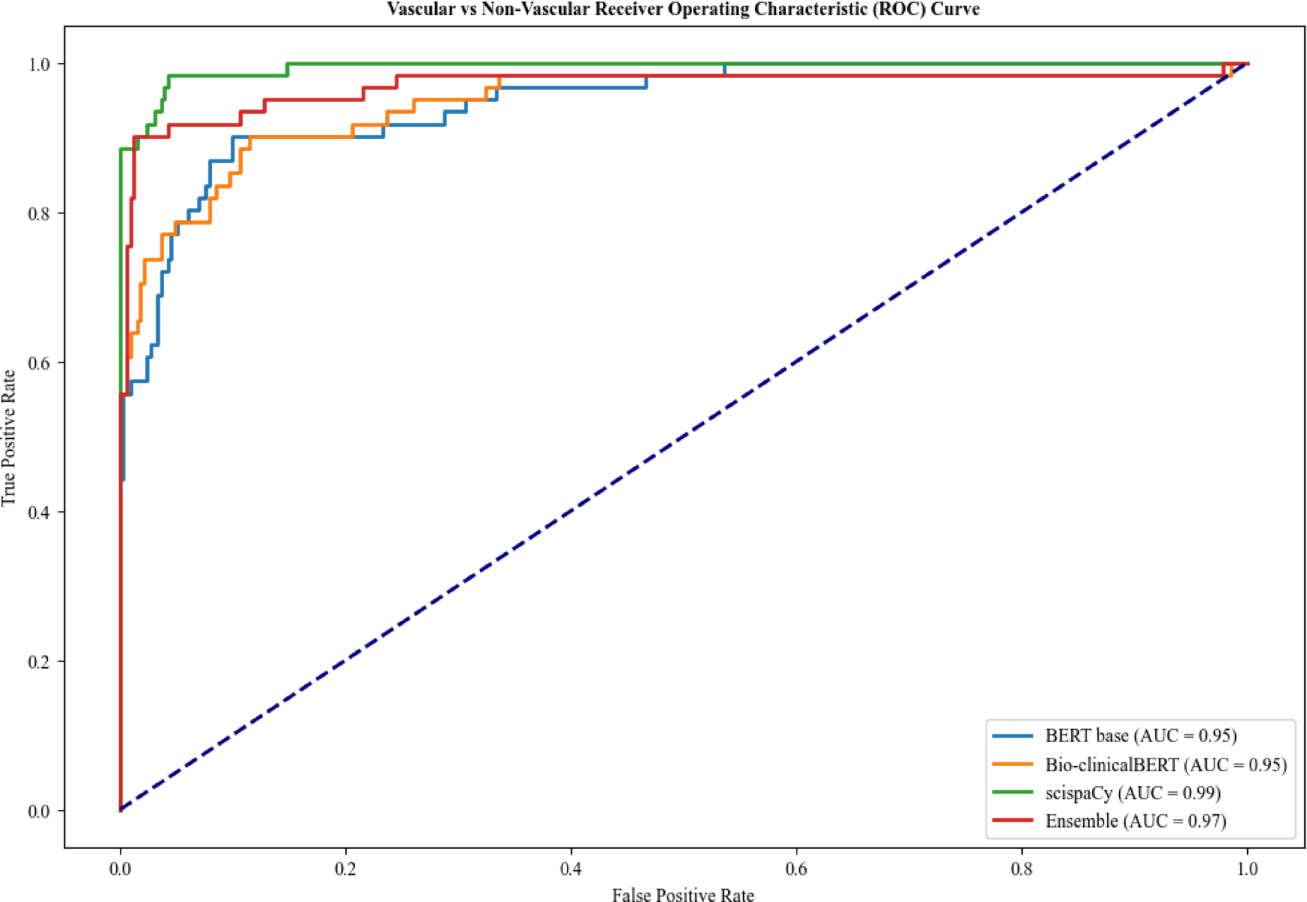
Comparison of receiver operating characteristic curves for Task 1 - Vascular vs. Non-Vascular classification.

For Task 1, the scispaCy and ensemble models had the highest overall accuracy of 0.97 and BERT-base had the lowest accuracy of 0.92 (Table 2). Bio-clinicalBERT demonstrated the lowest recall for classification of vascular cases at 0.70 compared to 0.79 for BERT-base and 0.89 for scispaCy and ensemble models, indicating a higher risk of missed cases. ScispaCy had the best discriminative ability with an AUC of 0.99 and Bio-clinicalBERT and BERT-base had the worst discrimination at 0.95 (Fig. 2).

**Table 2:** Table showing classification report for task 1 – classifying admissions for patients with acute vascular conditions using a threshold of 0.5. Evaluation set class distribution: vascular 15.6% (*n* = 61), Non-Vascular 84.4% (*n* = 330).

		Vascular			Non-Vascular		
Model	Accuracy	Precision (Vascular)	Recall (Vascular)	F1-Score (Vascular)	Precision (Non-Vascular)	Recall (Non-Vascular)	F1-Score (Non-Vascular)
BERT-base	0.92	0.74	0.79	0.76	0.96	0.95	0.95
Bio-clinicalBERT	0.94	0.88	0.70	0.78	0.95	0.98	0.96
ScispaCy	0.97	0.92	0.89	0.90	0.98	0.98	0.98
Ensemble	0.97	0.93	0.89	0.91	0.98	0.99	0.98

**Fig. 3:**
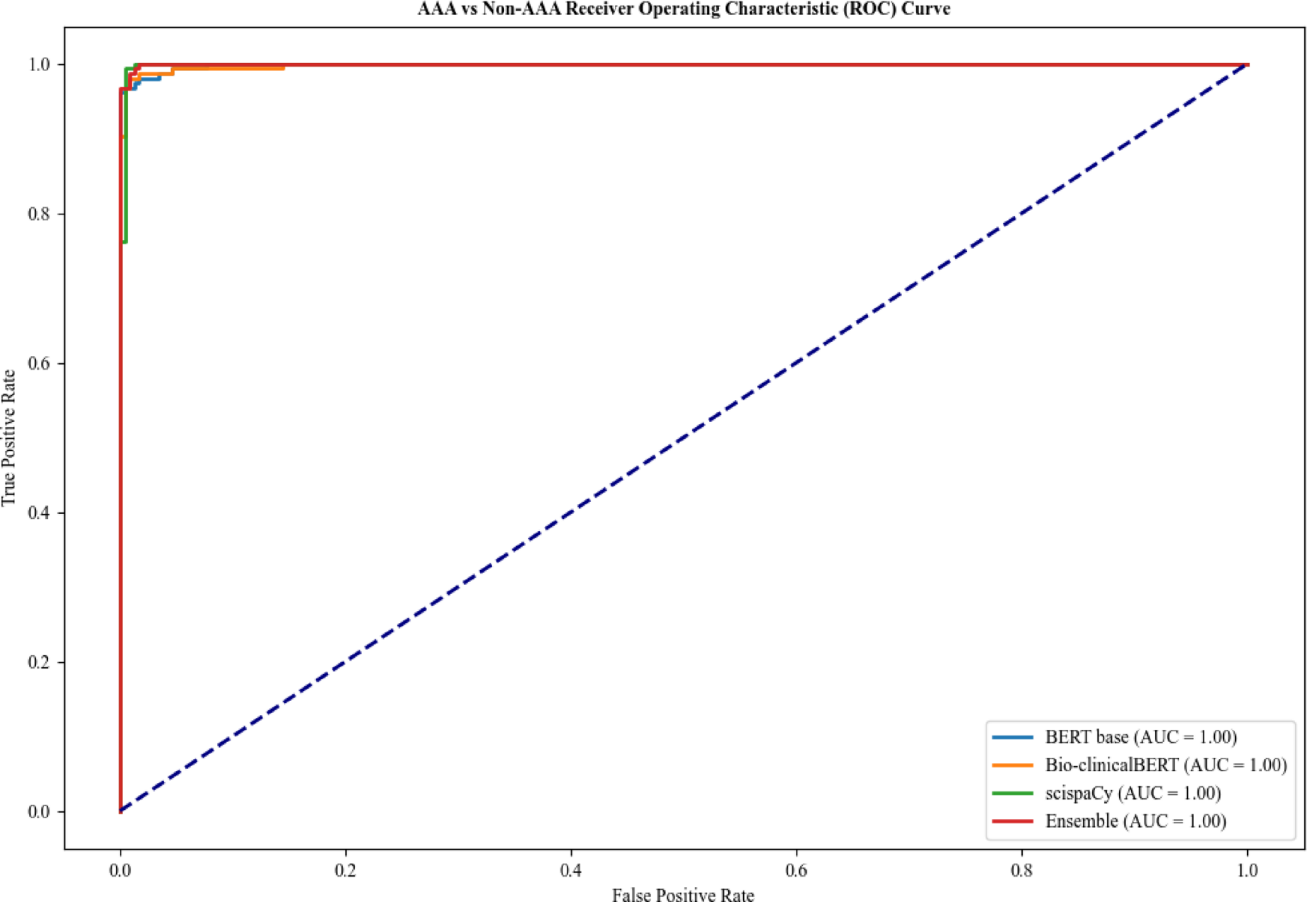
Comparison of receiver operating characteristic curves for Task 2 - AAA vs. Non-AAA classification.

For Task 2, all models performed very well across all metrics as shown in Table 3. The ensemble, scispaCy and Bio-clinicalBERT models showed the highest degree of accuracy of 0.99 with all models achieving an AUC of 1.00 (Fig. 3). BERT-base had a slightly worse recall of 0.97 when compared with the other models for AAA classification (0.98 and 1.00), indicating a marginally higher risk of missing AAA repair cases.

**Table 3:** Table showing classification report for task 2 – classifying admissions for patients who underwent AAA repair during their admission using a threshold of 0.5. Evaluation set class distribution: AAA 39.7% (*n* = 155), Non-AAA 60.3% (*n* = 235).

		AAA			Non-AAA		
Model	Accuracy	Precision (AAA)	Recall (AAA)	F1-Score (AAA)	Precision (Non-AAA)	Recall (Non-AAA)	F1-Score (Non-AAA)
BERT-base	0.98	0.99	0.97	0.98	0.98	0.99	0.99
Bio-clinicalBERT	0.99	0.99	0.98	0.98	0.99	0.99	0.99
ScispaCy	0.99	0.97	1.00	0.99	1.00	0.98	0.99
Ensemble	0.99	0.97	1.00	0.99	1.00	0.98	0.99

**Fig. 4:**
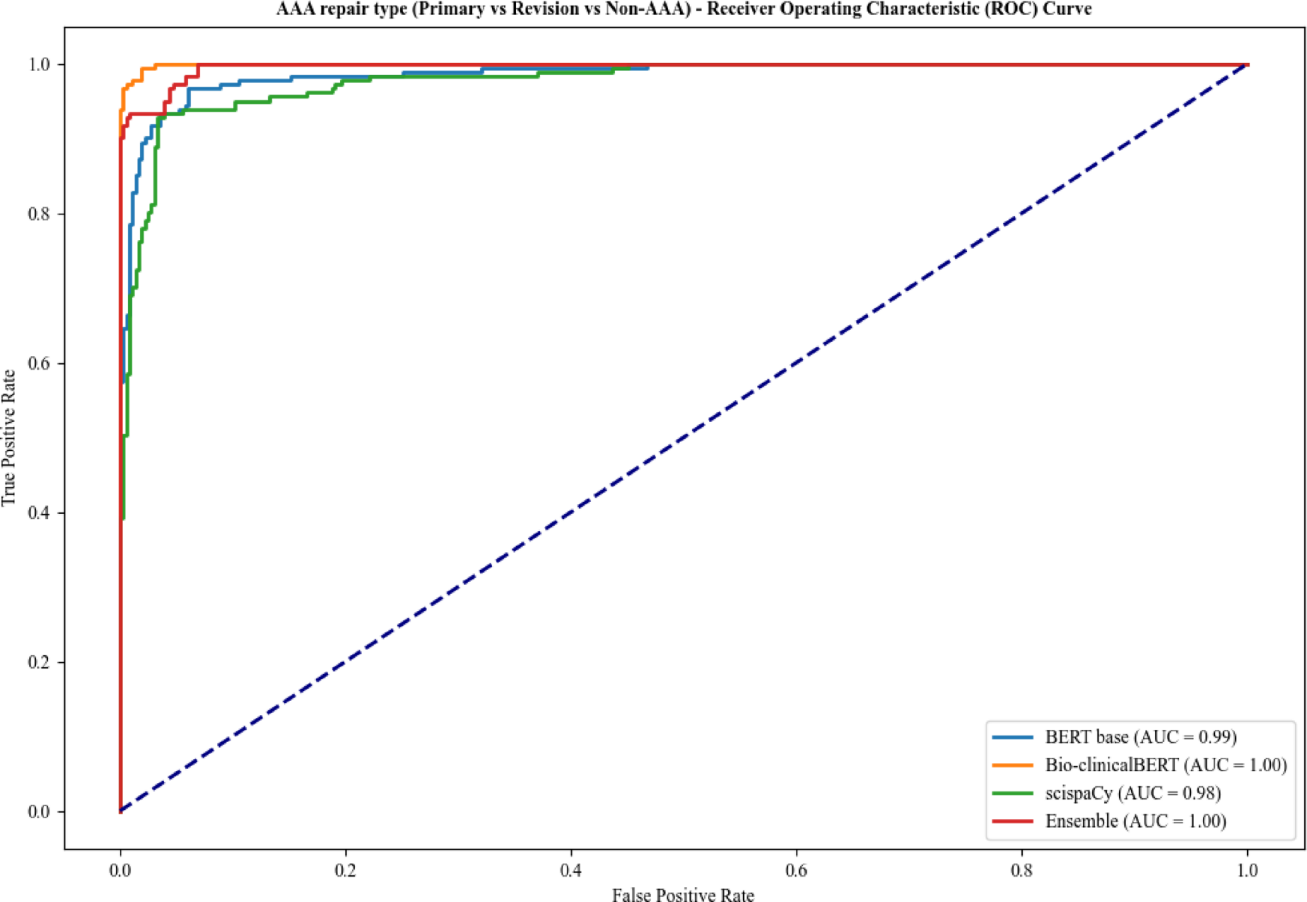
Comparison of receiver operating characteristic curves for Task 3 - Primary AAA repair vs. Revision AAA repair vs. Non-AAA classification.

For Task 3, Bio-clinicalBERT and ensemble models achieved an AUC of 1.00 (Fig. 4) with Bio-clinicalBERT displaying the best overall accuracy of 0.98 (Table 4). BERT-base had the lowest F1-score across all labels in Task 3 with Bio-clinicalBERT having the highest.

**Table 4:** Table showing classification report for Task 3 – classifying type of AAA repair undertaken during admission using a threshold of 0.5. Evaluation set class distribution: Non-AAA 43.3% (*n* = 84), Primary AAA 39.7% (*n* = 77), Revision AAA 17.0% (*n* = 33).

		Non-AAA			Primary AAA repair			Revision AAA repair		
Model	Accuracy	Precision	Recall	F1-Score	Precision	Recall	F1-Score	Precision	Recall	F1-Score
BERT-base	0.92	0.97	0.96	0.96	0.90	0.95	0.93	0.86	0.72	0.78
Bio-clinicalBERT	0.98	1.00	0.96	0.98	0.97	0.99	0.98	0.96	1.00	0.98
ScispaCy	0.93	0.99	0.93	0.96	0.93	0.93	0.93	0.79	0.92	0.85
Ensemble	0.93	0.99	0.93	0.96	0.93	0.94	0.94	0.82	0.92	0.87

Across the three classification tasks in this paper, Task 2 showed the most consistent highly accurate results across all models. The ensemble model consistently had the highest or joint highest F1-score across all classification tasks, apart from Task 3, demonstrating its robustness and reliability. BERT-base was the worst performing model across all tasks with the lowest F1-scores.

## Discussion

This multi-tiered NLP approach demonstrates a proof of concept for tackling classification tasks in healthcare, shown here by the identification and classification of AAA repairs from unstructured EHRs. The tiered approach allows for iterative refinement of the dataset, starting with broad classifications and progressively narrowing down to more specific categories. This method could be particularly useful in scenarios where traditional categorisation methods, such as ICD-10 codes, lack sufficient granularity for certain conditions, like acute limb ischaemia^24^. The integration of NLP models into existing EHR infrastructure has the potential to enhance data extraction and auditing processes across various medical specialties and other domains dealing with unstructured text data.

Several studies have applied NLP in cardiovascular medicine, but this is the first to specifically identify AAA repair patients and classify repairs as primary or revision^14–16,18–20,25,26^. The closest related work, Weissler et al., developed PAD-ML, an NLP model using an artificial neural network to identify peripheral arterial disease (PAD) from EHRs, achieving AUC 0.888^19^. While direct comparisons with PAD-ML are limited due to differing tasks, datasets, and metrics, our models show strong results in this context with our best-performing model, the scispaCy/Bio-clinicalBERT ensemble, achieving AUROCs of 0.99, 1.00, and 1.00 for identifying vascular patients, AAA repairs, and classifying repair types, respectively.

Other PAD-focused studies have used rule-based NLP approaches. Savova et al. employed a clinical Text Analysis and Knowledge Extraction System (cTAKES) with a PAD-specific dictionary, reaching 93% accuracy using radiology reports^18^. Afzal et al. used predefined PAD-related keywords and rules within clinical notes, achieving 92.7% accuracy^25^. While effective in structured settings, these methods relied on fixed dictionaries and rigid rules, limiting adaptability to new clinical terminology. In contrast, our machine learning models offer greater flexibility, utilising unstructured clinical text with minimal pre-processing. Additionally, McLenon et al. used a commercial NLP system with classifier and diameter-extraction modules to identify AAAs from radiology reports, achieving 95% precision and 88.5% sensitivity^16^. Our best performing models (scispaCy, Bio-clinicalBERT, BERT-base, and ensemble) for each task achieved better performance than these previous studies (accuracy 0.97–0.99), demonstrating that multiple modern NLP approaches can effectively handle complex clinical classification tasks.

The comparison between BERT-base, Bio-ClinicalBERT, and scispaCy highlights the impact of domain-specific pretraining on model performance. BERT-base, despite being a strong general NLP model, lacks exposure to medical terminology, leading to a lower ability to distinguish revision AAA repairs from primary procedures. ScispaCy, while computationally efficient, relies on static word embeddings and does not utilise deep bidirectional contextual embeddings like Bio-clinicalBERT. This makes it less effective at capturing nuanced differences between primary and revision AAA repairs, which often require an understanding of prior surgical history and procedural context spread across the EHR.

Bio-clinicalBERT’s superior performance in Task 3 may be attributed to its domain-specific pretraining on clinical corpora. The initial pretraining on MIMIC-III, PubMed and PubMed Central provides Bio-clinicalBERT with embeddings specialised in medical terminology^12,27^. During fine-tuning, these specialised embeddings are further adapted to the specific context of AAA repairs, potentially enabling the model to better distinguish between primary and revision procedures. The presence of medical terminology related to complications and reinterventions in its training corpus likely contributes to its improved ability to classify these cases correctly.

The ensemble model achieved competitive performance for Tasks 1 and 2 but required approximately twice the inference time of individual models while providing minimal performance gains. The ensemble matched scispaCy’s accuracy for Task 1 (0.97) and achieved identical performance to other models for Task 2 (0.99), but underperformed Bio-clinicalBERT for Task 3 (0.93 vs. 0.98 accuracy). Given the negligible accuracy improvements relative to computational overhead, individual models such as Bio-clinicalBERT for complex tasks and scispaCy for efficiency may be preferable.

The clinical significance of model performance is crucial for assessing its potential integration into automated patient identification workflows. The high recall of scispaCy and Bio-clinicalBERT models ensures that vascular and AAA cases are captured effectively, reducing the risk of misclassification and improving follow-up care. The high precision minimises false positives, ensuring that only true cases are flagged for registry inclusion.

The class imbalances observed across tasks (Tables 2, 3 and 4) reflect realistic clinical distributions but require careful interpretation of precision and recall metrics. The high precision for majority classes, particularly non-vascular cases in Task 1 (0.95–0.98), is partly attributable to their predominance in the evaluation set. However, model performance varied considerably, with Bio-clinicalBERT achieving perfect recall (1.00) for minority revision repairs while BERT-base struggled (0.72 recall), demonstrating that appropriate domain-specific pretraining can overcome class imbalance limitations. The observed class imbalances were not mitigated in this study but could be explored in future studies using techniques such as Synthetic Minority Oversampling Technique (SMOTE) or class-weighted loss functions.

With the development of increasingly complex generalised LLM models such as GPT-4, Claude, DeepSeek and larger clinical foundation models such as GatorTron, clinical NLP developers will need to balance model complexity, performance and interpretability to ensure that the model delivers on accuracy whilst maintaining computational efficiency and transparency in decision-making^28^.

There are limitations to the models developed in this paper which need to be considered in future development and implementation. Although the model was annotated by a single clinically trained annotator following a structured annotation guide, the use of a single annotator may introduce subjective bias and reduce the reliability of the annotated dataset. Future models intended for clinical use would benefit from multiple clinically trained annotators to establish formal inter-annotator agreement metrics (such as Cohen’s kappa).

The models were trained on a dataset from one hospital in the USA, thereby narrowing the breadth of medical language and style used in medical record keeping across the world. Although fundamental clinical practice is similar across countries, there were some differences in vascular practices identified in the US medical records compared to standard UK NHS practice, such as the management of acute diabetic foot infections by podiatry in the US EHRs. Given our proposed application to the UK National Vascular Registry, this represents a critical limitation that requires systematic validation before clinical deployment, as there may be subtleties in the records which negatively impact the generalisability of the models if used outside the original hospital context.

Future work will require a systematic approach to address these limitations and expand the model’s capabilities. External validation across geographically and demographically diverse settings will be essential to quantify how regional practices and language variations influence model performance. This validation process presents several technical challenges, including the development of standardised annotation guidelines, creation of automated data preprocessing pipelines to handle variations in EHR formatting, and implementation of secure data sharing infrastructure compliant with regional privacy regulations such as General Data Protection Regulation/HIPAA. Establishing data use agreements and governance frameworks between participating institutions will be crucial for successful multi-institutional collaboration.

The integration of other NLP tasks such as named entity recognition and/or span recognition would allow vascular pathology specific data (e.g. AAA diameter) to be identified and extracted from unstructured free text data, potentially enabling the automated data extraction and upload to national registries such as the NVR^16^. Modern frameworks like Langchain, when used with locally deployed models, could streamline the development of these extraction pipelines while maintaining data privacy and institutional control over sensitive clinical information. The development of such a pipeline would remove barriers such as administrative and time burden to allow a much broader data capture of patients, with the key challenge being ensuring the underlying models meet the high accuracy and consistency requirements of clinical data extraction. Additionally, integrating other data modalities such as imaging would allow AAA morphology to be captured in detail helping with predictive modelling^29^. The pipeline should also incorporate a clinician-in-the-loop mechanism that allows clinicians to provide feedback to the model and contribute to ongoing model refinement. Suggested initial implementation would involve a pilot study at a single UK NHS vascular unit to validate performance and establish workflows, before expanding to a regional vascular network for multi-institutional validation. This approach would establish standardised protocols and highlight key implementation issues prior to broader deployment. Beyond identifying AAA repairs, this framework could be adapted to identify other NVR-relevant patients such as those undergoing lower limb bypass or carotid endarterectomies. The technique could also be expanded to other medical speciality registries such as National Joint Registry or National Confidential Enquiry into Patient Outcome and Death, with standardised output formats ensuring seamless integration with each registry’s specific requirements.

This study presents a robust framework for developing NLP models to identify and classify AAA repairs from unstructured EHRs. The high accuracy achieved by our models demonstrates the potential for implementing these techniques in clinical data pipelines to improve auditing of AAA repairs at both local and national levels. Our work represents a significant step towards harnessing the untapped potential of unstructured EHR data across various medical specialties. By automating the extraction and classification of pertinent clinical information, NLP models can allow continuous surveillance as well as retrospective case finding, reduce administrative burden, increase data capture, and ultimately contribute to improved patient care and outcomes in vascular surgery and beyond.

## Electronic supplementary material

Below is the link to the electronic supplementary material.

Supplementary Material 1

## Data Availability

Models are available for download and use under GNU General Public License v3.0 at https://huggingface.co/danielcthompson with demonstration scripts available at https://github.com/dc-thompson/AAA_classification. The MIMIC IV dataset used for training is available at https://physionet.org/content/mimic-iv-note/2.2/. The underlying code and annotated dataset for model training used in this study is not publicly available but may be made available to qualified researchers on reasonable request to the corresponding author.
