# Supplementary material for: Natural Language Processing framework for identifying abdominal aortic aneurysm repairs using unstructured electronic health records
Source: Sci Rep. 2025 Jul 21;15:26388. doi: 10.1038/s41598-025-11870-6 (PMC12280078; doi:10.1038/s41598-025-11870-6)
Supplement: Supplementary file 1 — Supplementary Material 1 [file 41598_2025_11870_MOESM1_ESM.pdf]

Appendix 1

Seed terms – Task 1

```
{"label":"VASCULAR","pattern":"endarterectomy"}
{"label":"VASCULAR","pattern":"aneurysm"}
{"label":"VASCULAR","pattern":"bypass"}
{"label":"VASCULAR","pattern":"amputation"}
{"label":"VASCULAR","pattern":"vascular"}
{"label":"VASCULAR","pattern":"bypass"}
{"label":"VASCULAR","pattern":"endovascular"}
{"label":"VASCULAR","pattern":"arterial"}
{"label":"VASCULAR","pattern":"venous"}
{"label":"VASCULAR","pattern":"endovenous"}
```

Seed terms – Task 2

```
{"label":"AAA Repair","pattern":"AAA"}
{"label":"AAA Repair","pattern":"aneurysm"}
{"label":"AAA Repair","pattern":"abdominal aortic aneurysm"}
{"label":"AAA Repair","pattern":"aortic aneurysm"}
{"label":"AAA Repair","pattern":"EVAR"}
{"label":"AAA Repair","pattern":"TEVAR"}
{"label":"AAA Repair","pattern":"thoracic aorta"}
{"label":"AAA Repair","pattern":"thoracic aneurysm"}
```

Seed terms – Task 3

```
{"label":"AAA Revision","pattern":"endoleak"}
{"label":"AAA Revision","pattern":"revision"}
{"label":"AAA Revision","pattern":"redo"}
{"label":"AAA Revision","pattern":"revised"}
{"label":"AAA Revision","pattern":"relined"}
```
